# The Performances of Adlay (*Coix lacryma-jobi* L.) As Component of Agroforestry on Teak (*Tectona grandis* L.f.) Plantation

**DOI:** 10.21315/tlsr2024.35.2.5

**Published:** 2024-07-31

**Authors:** Titi Juhaeti, Nuril Hidayati, Ninik Setyowati, Albert Husen Wawo, Wahyu Widiyono

**Affiliations:** 1 Research Centre for Plant Conservation, Botanic Gardens and Forestry, National Research and Innovation Agency (BRIN), Cibinong, 16911 West Java, Indonesia; 2 Research Centre for Environmental and Clean Technology, National Research and Innovation Agency (BRIN), Kawasan Puspiptek Gedung 820, South Tangerang 15314, Indonesia

**Keywords:** Adlay, Cultivation, Growth, Production, Teak

## Abstract

Adlay is an edible high nutritious minor cereal. The research aimed to study the adlay performance when cultivated intercropped on young teak (*Tectona grandis* L.f.) plantation. The treatments were consisted of two factors that were arranged by factorial (3 × 3) in randomised completed block design with four replicates. The first factor was three fertilisation dosages of NPK (16-16-16), as 0 g/hole (F0), 2 g/hole (F2) and 4 g/hole (F4). The second factors were three planting space as 2 m × 2 m (PS2), 3 m × 3 m (PS3) and 4 m × 4 m (PS4). The parameter observed were vegetative growth which was consisted of plant height, the number of leaves, tillers and sub tillers, leaf chlorophyll content, grain production and biomass weight. The results revealed that fertilisation treatment were significantly affected the growth and production of adlay. On 12 weeks after planting, the F4 treatment produced the tallest plant, and the highest leaf number. The highest grain number/plant was achieved on the F2 treatment that was significantly different compared with the control (F0). The shading intensity due to the teak coverage significantly affected adlay growth and production. The PS4 treatment produced the highest number of leaves, tillers and grains. The combination of F2 and PS4 treatments resulted in the best growth and production. The PS2 treatment which has lower light intercepted by plants due to lower incident light intensity, resulting in a significant reduction in plant growth and production. It is suggested that adlay offers potency as a component of agro-forestry on the teak plantations.

**Highlights**

- The results revealed that fertilisation treatment significantly affected thegrowth and production of adlay.
- The shading intensity due to the teak coverage significantly affected adlaygrowth and production.
- The combination of NPK (16-16-16) fertilisers level of 2 g/plants and teakplanting space of 4 m × 4 m treatments resulted in the best growth andproduction of adlay.
- It is suggested that adlay offers potency as a component of agro-forestryon the teak plantations.

## INTRODUCTION

Adlay (*Coix lacryma-jobi* L.) is also known as *hanjeli* (West Java Indonesia), Chinese pearl barley, job’s-tears, belongs to Poaceae] family. Adlay is a highly nutritious minor cereal that has been consumed by people in some part of the world, including Indonesian people. Juhaeti (2016) and Handayani *et al*. (2019) stated that adlay has been cultivated in small scale throughout Indonesia (Java, Sumatra and Kalimantan islands). In Ciletuh Geopark Pelabuhan Ratu, Sukabumi Regency, West Java, local people developed a “Hanjeli Eco-Tourism Village”, where local and foreign tourists are provided interesting information about adlay cultivation, on-farm production, post-harvest processing and how to create various culinary based on adlay grain and flour (Juhaeti *et al*. 2020). In addition, adlay is potential as a wheat substitutes (Lim 2013).

Juhaeti (2016) found that adlay grain collected from West Java Indonesia is gluten-free that can be recommended as functional food for people that allergy to gluten or gluten intolerant. Adlay grain contains 1.95%–4.82% fat, 69.46%–74.79% carbohydrate, 8.51%–16.37% protein, 6.84%–8.36% dietary fibre, 6.84 mcg/100 g–27.63 mcg/100 g vitamin B6, 2.09 mcg/100 g–3.67 mcg/100 g vitamin B12 and 143.68 mg/100 g–174.41 mg/100 g vitamin E. Due to its nutritional value, adlay can be promoted as functional food and drink (Li & Corke 1999, Juhaeti *et al*. 2021). With this excellent nutritional and medicinal properties, adlay provides promising potencies that worths it for its exploitation and utilisation as staple crops (Nehal *et al*. 2015). *Coix lacryma-jobi* L. is also potential for ruminant feed through the application of feed processing technology such as hay, silage, amofer and complete feed (Mayulu *et al*. 2020).

Adlay is also known as an important minor herbal medicine in some Asian countries. A formulated adlay seed oil-drug is clinically applied to treat multiple cancers (Xi *et al*. 2016). Adlay can also be functioned as traditional oriental medicines, such as baked products, soups and tea, as well distilled liquor from flour or whole grain (Arora 1977). Adlay grain is proven to contain anti-tumour active compound (Numata *et al*. 1994). Some bioactive compounds in adlay seed could improve lipid metabolism, reduce liver fat accumulation, inhibit tumours, prevent cancer and provide protection against viral infection (Hidaka *et al*. 1992; Hung & Chang 2003; Chun *et al*. 2004, Chang *et al*. 2003; Yu *et al*. 2005). Yang *et al*. (2013) stated that adlay is also proven to prevent osteoporosis.

In Thailand, adlay production areas were located in the North and North-East parts. About 4,000 to 6,000 tonnes of adlay were exported to Japan and Taiwan each year from the areas (Grubben & Partohardjono 1996). Diao (2017) stated that in China, approximately 73,000 ha adlay cultivation area existed, with a grain yield of 0.22 million tonnes, where Guizhou is the largest producer, with a cultivation area of 32,000 ha.

In Indonesia, farmers cultivate adlay in small scales. As an under-utilised crop which has characteristics tolerant to drought and sub-optimum growing conditions, adlay can be recommended for marginal area coverage crop. Marginal land which have the sub-optimum environmental conditions such as low in soil fertility, low in water availability tend to increase year to year as well as high availability of shaded lands under plantations such as oil palm and teak which are reaching reaching 12.1 million ha. About 3%–4% of new plantation areas increase yearly, therefore it is potential for inter-cropped plants extension (Sopandie & Trikoesoemaningtyas 2011).

An effective intercropping strategy could overcome problems on food and feed supply (Gorne & Aradilla 2020). Intercropping is one alternative system to maximise the utilisation of farmlands. Average production of adlay under traditional cultivation was recorded at about 5.0 tonnes/ha with the range of 3.3 to 8 tonnes/ha (Suyadi *et al*. 2019). However, the harvest index of adlay was low, ranged on 37%–38%. This harvest index should be increased through increasing production per clump that can be achieved by fertilisation. Application of 200 kg/ha compound fertiliser was proven to increase the 1.000-grain weight by more than 15% and also increased grain yield by more than 25% (Suyadi *et al*. 2019). It is also important to study the agro-physiological properties of adlay as a basic for its optimal cultivation on marginal shading area. Juhaeti *et al*. (2020) showed that some of West Java adlay germplasms were tolerant to 25%–50% low light intensity. The research aimed to evaluate the agronomical performances of some Indonesian adlay cultivars as an agro-forestry component, cultivated under different planting spaces of teak stands with various doses of NPK fertilisation.

## MATERIALS AND METHODS

The present research was conducted on teak plantation located at The Research Center for Biology, Indonesian Institutes of Sciences, Cibinong Science Centre, Bogor, West Java, Indonesia. The treatments were arranged by factorial (3 × 3) in randomised completed block design with four replicates. The first factors were three fertiliser dosages of NPK (16-16-16), as 0 g/hole (F0), 2 g/hole (F2) and 4 g/hole (F4). The second factors were three planting spaces, as 2 m × 2 m (PS2), 3 m × 3 m (PS3) and 4 m × 4 m (PS4). Teak plantation was 2.5 years old, the average of plant height was 250 cm, and the stem diameter was 10 cm.

Planting bed preparation was done first of all by determining the planting area under teak plantation, clearing the weed in the area, ploughing and tilling the soil to perform planting bed and preparing the planting holes. The number of planting bed were 3 fertiliser × 3 teak planting space × 4 replicate = 36 planting beds. Each planting bed consisted of 60 planting holes. The plants as replicates in this experiment was 3 fertiliser × 3 teak planting space × 4 replicates × 2 plant = 72 plants. Seed planting was done directly with three seeds within a planting hole at about 3 cm depth, at 60 cm × 60 cm planting space. At one week after planting (WAP), the number of plants per hole was reduced to one plant. The NPK (16-16-16) fertilisation was applied twice according to the mention doses above, respectively on one and three weeks after planting (WAP).

The observations and data recording on plant growth were conducted every week starts on 2 WAP. The variables observed included the growth at vegetative stage which consisted of plant height (measured from the soil surface to the longest leaf tip), the number of leave blade (weekly count of the leaf blade increment), and the number of tiller (newly stem emerged from the soil surface attached to each main stem), sub-tiller (the branches of the main stem) observed on 2 to 12 WAP. Observations on leaf greenness which represent the chlorophyll content (mentioned below) was conducted on 7 WAP. Observations on generative phase consisted of flowering time, seed production, and biomass weight conducted at harvest time. The grain was harvested when approximately 90% of panicles had been matured, which was indicated by brownish color at about 23 WAP.

The microclimate on teak plantation was measured using Digital Thermo-hygrometer AS ONE TH-321 (Corona) for temperature and humidity and Lux-meter (LUXOR) for light intensity.

**Figure 1:**
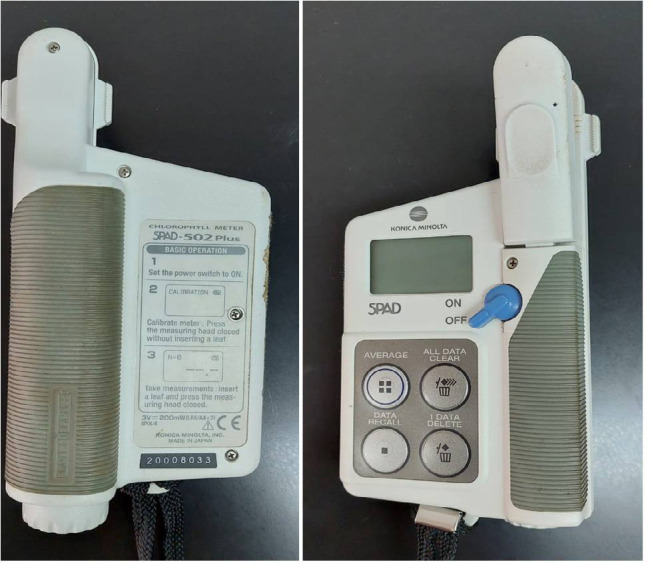
SPAD 502plus Chlorophyll meter apparatus (Konica Minolta, https://www.konicaminolta.com/instruments/download/catalog/color/pdf/spad502plus_catalog_eng.pdf).

The chlorophyll content of the leaves was determined by Chlorophyll meter SPAD-502Plus apparatus (Fig. 1). SPAD-502Plus determines the relative amount of leaf chlorophyll content by measuring the absorbance of the leaf in two wavelength region which are the blue (400 nm–500 nm) and the red (600 nm–700 nm) regions. Using this two absorbances, the matter calculates a numerical SPAD value which is proportional to the amount of leaf chlorophyll (Konica Minolta SPAD-502Plus). According to Jiang *et al*. (2017) the correlation of SPAD value and total chlorophyll content were significantly correlated in tomato leaves (*r* = 0.869). The data proved that SPAD-502 plus can become an effective tool for rapid and non-destructive determination of leaf chlorophyll content. Meanwhile, according to Villa *et al*. (2022), estimating chlorophyll content using SPAD-502 Plus was directly correlated with the absolutes content in okra leaves.

Data analysis in this experiment was conducted using SAS 9.1 software with a two-way analysis of variance (ANOVA) at a 5% confidence level. Further significance testing is performed using the DMRT test at the 5% level when there was a significant effect between treatments.

## RESULTS

### The Microclimate on Different Teak Planting Space

The observation showed the differences of microclimate among teak planting spaces especially on light intensity. The light intensity decreased as teak planting space decreased. The highest light intensity was obtained at 4 m × 4 m teak planting space which was 93,700–115,700 lux, followed by 3 m × 3 m planting space 75,700–91,600 lux, and the lowest was at 2 m × 2 m teak planting space 54,300–61,400 lux. Both temperature and air humidity among teak planting spaces showed no differences. The temperature ranged 31.4°C–32.8°C, and air humidity was 69.3%–75.7%.

### Plant Height

**Figure 2:**
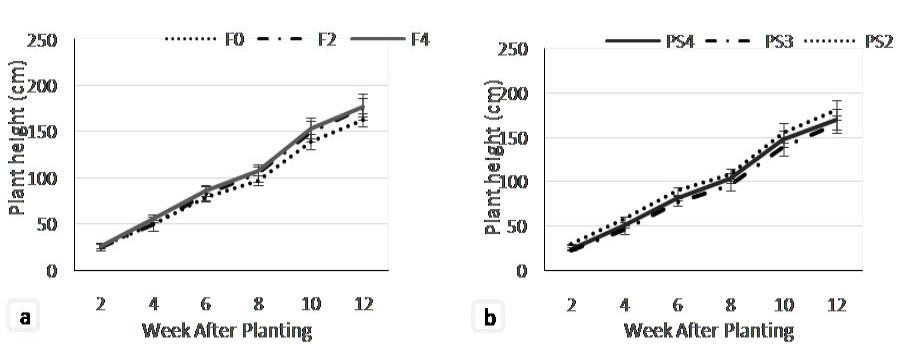
Plant height on (a) fertiliser dosage; and (b) teak planting space treatments at 2 to 12 weeks after planting.

The plant height of adlay as an effect of fertilisation treatments increased as the increasing plant aged. At the beginning of the growing stage (two weeks after planting, WAP), the plant height looked uniform ranging from 25.29 cm–26.19 cm. Furthermore, the plant height showed a significant growth starting from 4 WAP (55.85 cm) in F4 significantly different from F2 (50.46 cm) and F0 (48.70 cm) treatments (Fig. 2a). At 6–12 WAP, the plant height on F4 treatments showed the highest value followed by F2 treatment, that was significantly different with control. The lowest value of plant height was on an F0 treatment (163.05 cm).

Teak planting space significantly affected adlay plant height. Fig. 2b showed the pattern of plant height increment of adlay in all planting spaces. At 2–6 WAP, the PS2 treatment showed the highest value which was significantly different with PS3 and PS4. At 8–12 WAP, the plant height on PS2 and PS4 were significantly different with PS3. The highest plant height was obtained on PS2 treatment (180.48 cm), while the lowest was on PS3 treatment (165.38 cm).

**Figure 3:**
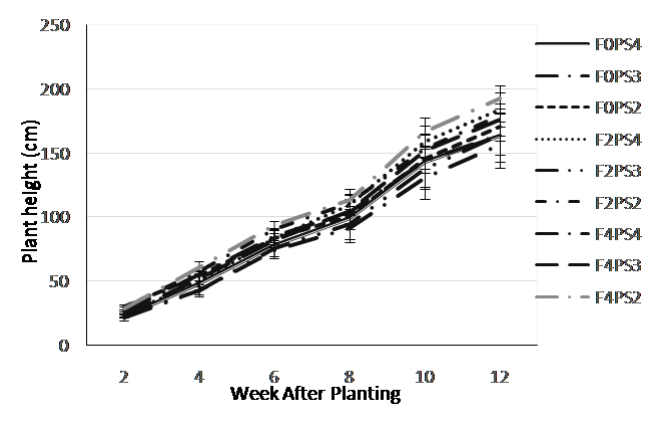
Plant height on combination of fertiliser dosage and teak planting space treatments at 2–12 WAP.

There were an interaction effects of fertilisation and teak spacing treatment on adlay plant height (Fig. 3). At the early of growth stage (2 WAP) the tallest plants occurred on F0PS2 treatment (30.2 cm) and on 3 WAP was detected on F2PS2 treatment (43.4 cm). Furthermore, until the age of 12 MST, the F4PS2 treatment consistently showed the highest value (192.143 cm) while the lowest was observed on F0PS3 treatment (156.143 cm).

### Number of Leaves

**Figure 4:**
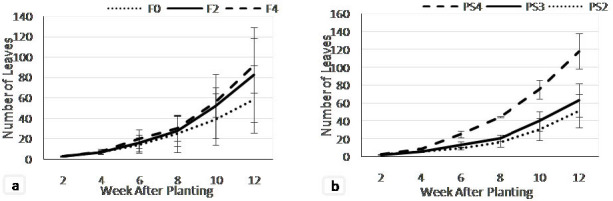
Leaves number on (a) fertiliser dosages; and (b) teak planting space treatments at 2–12 weeks after planting.

The single factor of fertilisation treatment (Fig. 4a) did not show differences on leaves number at the early growth stage (2WAP) that ranged 2.57–2.73 leaves. However, significant differences was detected at 4–12 WAP. The F4 showed the highest leaves number, followed by F2 and F0 treatments.

The teak planting space (Fig. 4b) also significantly affected the increment of adlay leaves. The significant effect was detected at 4 weeks onward where the PS4 treatment showed the highest leaf number that was significantly different from the PS3 and PS2 treatments.

**Figure 5:**
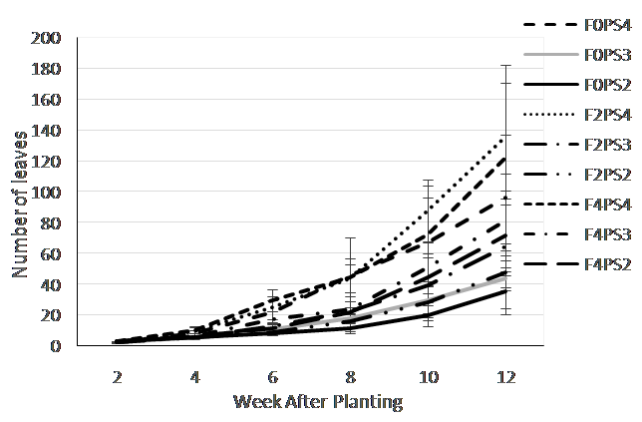
Leaves number on combination of fertiliser dosage and teak planting space treatments at 2–12 WAP.

The combination of fertilisation and teak planting space treatments (Fig. 5) were affected the number of adlay leaves. At the beginning of growth stage (2 WAP) the highest leaves number obtained from the F2PS2 (2.9), however at 2–6 WAP, the highest leaves number occurred on F4PS2 treatments. Meanwhile at 10 up to the end of the observation, the F2PS4 consistently produced the highest number of leaf (135.7), followed by F4PS2, while the lowest was F0PS2 treatment (35.3).

### Number of Tillers

**Figure 6:**
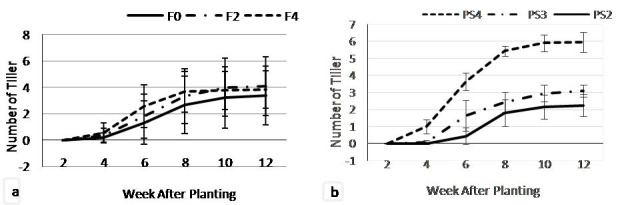
Tillers number on (a) fertiliser dosages; and (b) teak planting space treatments at 2–12 weeks after planting.

The adlay tillers emerged on the soil surface growing from the side of adlay main stem, started at 4 WAP. At 4 WAP, F4 treatment showed highest number of tiller (0.53) which was not significantly different with F2 (0.33) but significantly different with F0 (0.20). F4 treatment consistently produced the highest number of tillers up to 8 WAP. However, on 10–12 WAP, the fertilisation treatments did not show significant on number of tiller (Fig. 6a).

Fig. 6b showing that there was an indication that the more specious planting distance of teak affected in the more tiller production of adlay plant. The adlay grown under the widest teak planting space of 4 m × 4 m (PS4) produced the most tillers (5.95) that was detected at 4 WAP, significantly different from the planting space of 3 m × 3 m (PS3) and planting space of 2 m × 2 m (PS2). Meanwhile, the number of tillers on the PS3 (3.1) was not significantly different from the PS2 treatment (2.24).

**Figure 7:**
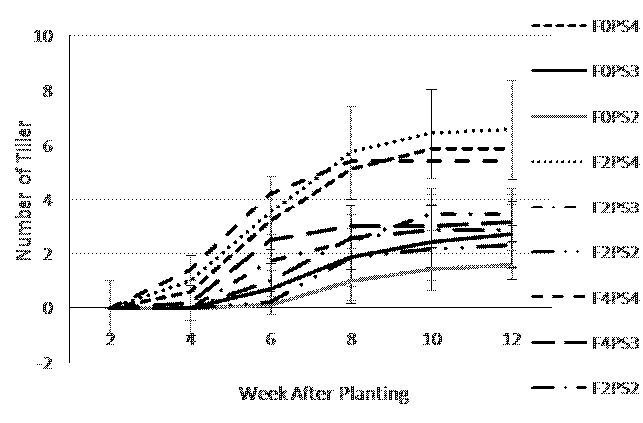
Tillers number on combination of fertiliser dosage and teak planting space treatments at 2–12 WAP.

The combination of fertilisation and teak planting space treatment (Fig. 7) showed the significant effect to the number of tillers variable. The adlay tiller started to emerge on four WAP on F4PS4 (1.4), F2PS4 (1.0), F0PS4 (0.6) and F4PS3 (0.2). On 8 to 12 WAP, the F2PS4 showed the highest number of tillers, followed by the F0PS4 and F2PS4 treatments. The smallest number of tillers was obtained from the F0PS2 treatment.

### Chlorophyll Content

**Figure 8:**
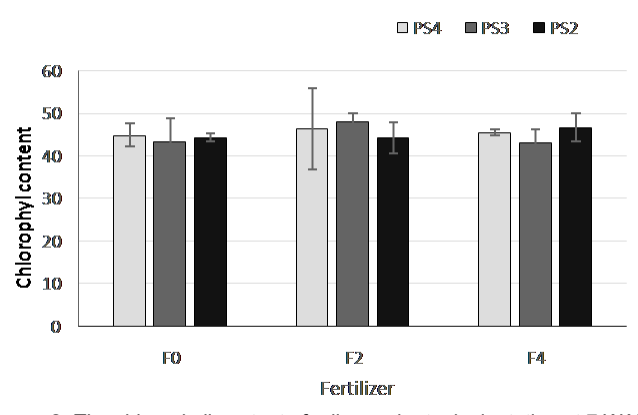
The chlorophyll content of adlay under teak plantation at 7 WAP.

Physiologically leaf chlorophyll content is related with the light intensity and nutrient, especially N availability, therefore in this study leaf chlorophyll content was examined as dependent variable to light intensity under different planting distances and N availability under different fertiliser treatments. On 7 WAP, the leaf chlorophyll content in all fertilisation treatment ranged 44.22–46.3 SPAD. Fertiliser treatment of 2 g/planting hole (F2) and 4 g/planting hole (F4) tent to show higher of leaf chlorophyll content compared to control (F0, no fertilised applied) although statistically not significant. The teak planting space treatment also did not significantly affect the adlay chlorophyll content (Fig. 8). The chlorophyll content in PS3 showed the highest value (45.6 SPAD), followed by PS2 (45.2) and PS4 (44.9 SPAD).

### The Biomass Production at Middle Stage of Vegetative Stage

The biomass production (shoot and root fresh weight) that was observed on 7 WAP as representative biomass production at the middle of vegetative stage to determine its potential for forages. Fertilisation significantly affected biomass production, where the F4 which was considered the highest dosage of NPK fertiliser showed the highest biomass production (Table 1). Similarly, teak planting space also significantly affected biomass production, where the PS4 which was considered the widest distances of teak and thus the highest level of light intensity, produced the highest biomass production.

There was a significant interaction effect between fertilisation and teak spacing treatment on biomass production that was observed on 7 WAP (Table 2). In all teak planting space, the increased of fertiliser dosage resulted in increased of biomass production. The combination of F4PS4 treatment showed the highest shoot fresh weight (168.7 g) and root wet weight (21 g) that was significantly different from other treatments.

### The Adlay Performance at the End of Vegetative Stage

The vegetative stage observation was terminated on 12 WAP, the time when vegetative stage ending and generative stage starting. The single effect of fertilisation treatment showed that the F4 and F2 treatments significantly affected the adlay growth (as seen on the high value of plant height, leaves number, and sub-tillers number), that was significantly different from the control (Table 3). The results of SAS analysis showed an interaction effect among fertilisation treatment and teak planting space (Table 4). The vegetative growth of adlay increased with the increasing fertiliser dosage in all teak planting space treatments. The best growth was achieved especially on the variables of the number of leaves, tillers and sub-tillers.

Fertilisation treatment was able to improve the growth of adlay even under most dense planting space of teak (PS2). In F0 (no fertilisation applied), the number of leaves in PS2 was 35.9 sheets, increased in the F2 treatment (47.57) and the highest was in the F4 treatment (71.86). The number of tillers in the PS2 treatment also increased from 1.57 in the F0 to 2.29 and 2.86 in the F2 and F4 treatments. The number of sub-tillers was increased from 4.86 in F0 to 7.29 and 10.43 in F2 and F4 treatments (Table 4).

The interaction effect of fertilisation and teak planting space treatments was occurred on PS3 and PS4. The number of leaves, tillers and sub-tillers were increased with the increasing of fertiliser dosage. The highest leaf numbers was obtained on F2PS4 (135.71) that was not significantly different from F4PS4 (122.00) and was significantly different with other treatments. The F2PS4 treatment showed the highest number of tillers (6.57), that was not significantly different from F4PS4 (5.43) and F0PS4 (5.86). In the sub-tiller variable, the F2PS4 treatment showed the highest number (22.00) that was not significantly different from F4PS4 (17.43) and was significantly different from other treatments.

### Flowering and Harvesting Time

The flowering stage of adlay started on 11 WAP, and seed maturity occurred on 22 WAP. Harvest was done at 23 WAP when 90% of panicles had been matured, which was indicated by the panicle becoming dry and brownish, meanwhile, the shoot was still fresh enough on both of leaf and stem.

The effects of fertiliser and planting space treatments on harvestable variables were observed, included the number of tillers, the number of sub-tillers, the shoot fresh weight, grain number and the weight of 100 grains. The results revealed that fertilisation treatment showed no significant effects on the number of tillers and 100-grains weight, although the real data tended to increase as increasing of fertiliser applied (Table 5). Fertilisation significantly affected the number of side shoots, shoot fresh weight and number of grains. The F4 treatment produced the highest number of sub-tillers and shoot fresh weight which were not significantly different with F2, but significantly different with F0. On the number of grains, the F2 showed the highest value which was not significantly different with F4 but was significantly different with F0.

The teak planting space significantly affected all of the variable observed except weight of 100 grain. The PS4 treatment showed the highest values on all observed variables, while the PS2 treatment showed the lowest values (Table 5). There was an interaction between fertilisation and teak planting space on all of variables observed at harvest time (Table 6). Similar as the results of observations at 12 WAP, the fertilisation showed a good effect on the number of tillers, the number of sub-tiller, the wet weight of the crown and the number of seeds in all conditions of teak space. The F2PS4 treatment showed the highest number of tillers (8.0), the number of side shoots (63.37) and the number of seeds (1917), were not significantly different from F4PS4 and F4PS3. In the shoot fresh weight variable, the highest yield was obtained from the treatment of F4PS4 (1154.7 g) which was not significantly different from F4PS4 (1107 g) and F4PS3 (1036.8 g).

## DISCUSSION

**Figure 9:**
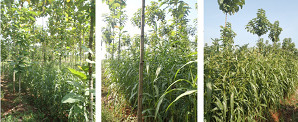
The physical performances of adlay under different teak planting spaces at 10 weeks after planting.

The physical performance of adlay as a result of the effect of fertilisation and teak planting space treatment showed on Fig. 9. Overall, adlay was able to grow in all teak planting spaces although differences in growth and production were detected. The adlay cultivated in PS4 showed the best growth and production, followed by PS3. The growth of adlay planted on PS2 was stunted and produced a few grains. The shading intensity as an effect of different teak planting space significantly affected adlay growth and production. Teak planting space treatment that related to different light intensity showed a significant effect on plant growth and production. The closer the spacing of teak, the light intensity that reaches the crops under main stands of agro-forestry system is decreases. Its means that shading intensity is increased. The result consisted with Juhaeti *et al*. (2020) that adlay growth and production decreased with the increasing of shading.

The PS2 treatment (light intensity of 54,300–61,400 lux), showed the highest value in plant height which was significantly different compared with PS3 (light intensity of 75,700–91,600 lux) and PS4 (light intensity of 93,700–115,700 lux). Whereas in the variable leaves and tillers number, the PS4 treatment showed the highest value and significantly different with PS3 and also PS2 (Table 3). It is obvious that PS4 with light intensity of 93,700–115,700 lux, that was the highest light intensity level in the treatment allowed more radiation intercepted on plant canopy and thus resulted in more vigourous growth. The increase of light intensity reaching the plants makes the plants grow vigour. The adlay growth and production showed better performances on the high light intensity, as seen in that PS4 treatments showed the highest growth and production.

Adlay was known as the C4 plant (Handayani *et al*. 2019) when the leaves have higher radiation, water, and nitrogen use efficiencies compared to the C3 species (Zehong *et al*. 2015). The decrease in growth and production of adlay planted on low light intensity also occurred in other C4 plants, such as sorghum and foxtail millet. On sorghum cultivation, Novitasari *et al*. (2016) recommended cultivating in the early stage of an agroforestry area (0%–25% shading) and not recommended in the middle (25%–50%) and advanced stage (more than 75%) due to the decrease of light intensity that reaching the land surfaces of middle and advanced agroforestry.

The biomass accumulation was dependent on radiation use efficiency and light interception (Sankalpi & Thomas 2014). The light intensity is high and related to the plant photosynthesis process. Under normal conditions, plants will allocate energy and nutrient for their growth and production, meanwhile, under stressful conditions, plants will use much more energy and nutrient for their survival (Prasch & Uwe 2015), finally decreasing growth and production. Nandini and Sridhara (2019) stated that low light penetration on adlay canopy reduced its photosynthesis and plant production. So, the lower light intensity (as a shading effect on agroforestry cultivation) is an abnormal condition for adlay growth and production.

The chlorophyll content in PS3 showed the highest value (45.6 SPAD), followed by PS2 (45.2) and PS4 (44.9 SPAD). It is consistent with Juhaeti *et al*. (2020) that stated the increasing of chlorophyll content under light shading and then become decreased under heavy shading condition.

Shorter teak planting space lowered light intensity reaching adlay shoot. So, the growth and production of adlay decreased significantly. It is accordance with Parande *et al*. (2019) that showed biomass and grain yield of adlay reduced by 61% with increasing shading intensity compared with no shading. The C4 plant has photosynthesis high energy costs and low plasticity compared with C3 in low light environments (Balasaheb *et al*. 2018). Yuan *et al*. (2017) showed that on foxtail millet (C4 plant) the grain fresh mass per panicle, yield, photosynthetic pigment contents, net photosynthetic rate, stomatal conductance, effective quantum yield of PSII photochemistry and electron transport rate decreased with the increase of shading intensity. Shading (low light intensity) also changed a double-peak diurnal variation of photosynthesis to a one-peak curve. The lower yield of foxtail millet was caused mainly by a reduction of grain mass assimilated, a decline in chlorophyll content, and a low photosynthetic rate due to low light during the grain filling stage. Reduced light energy absorption and conversion, restricted electron transfer and reduced stomatal conductance might cause a decrease in photosynthesis.

Fertilisation significantly affected plant growth and production. The F4 which was considered the highest dosage of NPK fertiliser, showed the highest biomass production. The fertilisation treatment of 2 g/planting hole was quiet enough to increased plant growth and production as F2 not significantly different compared to F4 treatment. This study demonstrated that adlay cultivation does not require a lot of fertiliser to achieve optimal growth. This finding is in line with Gorne and Aradilla (2020) statement that adlay can tolerate poor soil condition, grows well in sloping areas and is resilient to pests and diseases, produces a large amount of biomass that can be used as mulch to mitigate soil erosion on slopes, hence, it is a suitable for sustainable agriculture. Juhaeti (2015) also stated that adlay cultivation does not require a lot of fertiliser, a fertiliser dose of 2:1:1 g of urea:SP36:KCl (g/polybag) is sufficient for increase the productivity of adlay. Meanwhile, on intercropping cultivation, fertilisation scheme significantly influenced the height, number of tillers hill-1, fresh herbage yield and dry matter yield of adlay ratoon compared to without fertilisation (Gorne 2020). Fertilisation scheme significantly affected the days to flowering and maturity, vegetative tillers, plant height, number of productive and unproductive tillers, panicle length, herbage and grain yields of adlay intercropped with napier grass (Gorne & Aradilla 2020).

The high biomass production of adlay on vegetative stage showed the adlay potential for fodder. Mayulu *et al*. (2020) stated that adlay biomass is potential for fodder, the dry matter digestibility (DMD) of leaves was 59.56%, the organic matter digestibility (OMD) was 38.22%; DMD of stem was 51.77% and OMD of stem was 34.41%.

## CONCLUSION

The results suggested that both fertilisation and teak planting space treatments significantly affected adlay growth and production. Although fertilisation showed a positive effect, but adlay did not seem require large amount of fertiliser. An application of 2 g of NPK (16-16-16)/planting hole seems sufficient enough for adlay. The light intensity significantly was proven to affect adlay growth and production. The best adlay growth was achieved in lower shading intensity (PS4). The fertilisation treatments in lower light intensity (PS3 and PS2) can improve adlay growth and production. The optimal adlay growth and production were achieved on a combination of 2 g fertiliser and 4 m × 4 m teak planting space treatments.

## Figures and Tables

**Table 1:** The adlay biomass on each of fertilisation dosages an teak planting space treatments at 7 weeks after planting.

Treatment	Shoot fresh weight (g)	RootfFresh weight (g)
NPK fertilisation (g/planting hole)		
F0	57.08 ± 16.77 b*)	6.09 ± 0.99 b
F2	67.08 ± 20.09 b	5.34 ± 2.28 b
F4	107.87 ± 28.68 a	10.30 ± 1.50 a
Teak planting space (m)		
PS4	116.09 ± 35.87 a	13.28 ± 2.34 a
PS3	64.42 ± 12.79 b	5.17 ± 1.17 b
PS2	51.51 ± 16.88 c	3.29 ± 1.26 c

*Note*:

*) Means followed by the same letter in the same column indicated not significantly different by DMRT at the level of 5%.

**Table 2:** The adlay biomass on combination of fertiliser dosages and teak planting space treatments at 7 weeks after planting.

Interaction of fertiliser × planting space	Shoot fresh weight (g)	Root fresh weight (g)
F0PS4	87.13 ± 23.88 b*)	10.63 ± 1.45 b
F0PS3	49.23 ± 12.30 bc	4.87 ± 1.06 cde
F0PS2	34.87 ± 14.12 c	2.77 ± 0.47 e
F2PS4	92.47 ± 42.90 b	8.23 ± 4.24 bc
F2PS3	54.57 ± 3.62 bc	4.07 ± 0.81 de
F2PS2	54.20 ± 13.74 bc	3.73 ± 1.80 de
F4PS4	168.67 ± 40.83 a	20.97 ± 1.34 a
F4PS3	89.47 ± 22.44 b	6.57 ± 1.66 cd
F4PS2	65.47 ± 22.78 bc	3.37 ± 1.50 de

*Note*:

*) means followed by the same letter in the same column indicated not significantly different by DMRT at the level of 5%.

**Table 3:** The adlay growth on each of fertilisation dosages an teak planting space treatments at 12 weeks after planting.

Treatment	Plant height (cm)	Number of leaves	Number of tiller	Number of sub-tiller
NPK fertilisation (g/planting hole)				
F0	163.05 ± 6.93 b*)	58.43 ± 33.17 b	3.38 ± 2.22 a	7.29 ± 4.56 b
F2	175.91 ± 10.21 a	82.71 ± 46.71 a	4.10 ± 2.22 a	13.33 ± 5.36 a
F4	177.19 ± 14.21 a	91.71 ± 26.65 a	3.81 ± 1.41 a	13.86 ± 8.07 a
Teak planting space (m)				
PS4	170.29 ± 11.88 b	118.05 ± 19.94 a	5.95 ± 0.58 a	17.24 ± 9.51 a
PS3	165.38 ± 9.75 b	63.24 ± 18.91 b	3.10 ± 0.36 b	9.71 ± 5.97 b
PS2	180.48 ± 11.12 a	51.57 ± 18.61 b	2.24 ± 0.64 b	7.52 ± 2.51 b

*Note*:

*) Means followed by the same letter in the same column indicated not significantly different by DMRT at the level of 5%.

**Table 4:** The adlay growth on combination of fertiliser dosages and teak planting space treatments at 12 weeks after planting.

Interaction of fertiliser × planting space	Plant height (cm)	Number of leaves	Number of tiller	Number of sub-tiller
F0PS4	163.00 ± 18.01 cd*)	96.43 ± 40.69 bc	5.86 ± 2.61 a	12.29 ± 7.20bcd
F0PS3	156.14 ± 17.74 d	43.57 ± 22.68 de	2.71 ± 1.25bc	4.71 ± 4.61 d
F0PS2	170.00 ± 10.89 bcd	35.29 ± 10.69 e	1.57 ± 0.53 c	4.86 ± 1.86 d
F2PS4	184.00 ± 13.28 ab	135.71 ± 35.16 a	6.57 ± 1.81 a	22.00 ± 8.45 a
F2PS3	164.43± 16.30 cd	64.86 ± 27.08 cde	3.43 ± 0.79 b	10.71 ± 6.13bcd
F2PS2	179.29 ± 4.86 abc	47.57 ± 11.31 de	2.29 ± 0.76bc	7.29 ± 1.50 cd
F4PS4	163.86 ± 20.65 cd	122.00 ± 60.21 ab	5.43 ± 1.51 a	17.43 ± 12.87ab
F4PS3	175.57 ± 12.58 abc	81.29 ± 30.41 cd	3.14 ± 0.69bc	13.71 ± 7.18bc
F4PS2	192.14 ± 10.57 a	71.86 ± 23.77 cde	2.86 ± 1.35bc	10.43 ± 4.16bcd

*Note*:

*) Means followed by the same letter in the same column indicated not significantly different by DMRT at the level of 5%.

**Table 5:** The adlay growth and production on each of fertilisation dosages an teak planting space treatments at 23 weeks after planting.

Treatment	Number of tiller	Number of sub-tiller	Shoot fresh weight (g)	Number of grain/plant	Weight of 100 grain (g)
NPK fertilisation (g/planting hole)					
F0	5.1 ± 1.1a*)	33.3 ± 4.3b	577.6 ± 101.5b	978.1 ± 69.4b	13.1 ± 1.0a
F2	5.4 ± 0.96a	42.2 ± 8.6ab	832.5 ± 151.1a	1338.3± 180a	13.6 ± 1.2a
F4	6.3 ± 2.4a	47.2 ± 13.8a	915.5 ± 220.8a	1253.0 ± 169a	18.2 ± 7.9a
Teak planting space (m)					
PS4	7.6 ± 1.1a	56.9 ± 8.3a	1008.0 ± 146.7a	1708.9 ± 103.3a	12.5 ± 1.2a
PS3	5.4 ± 1.9b	43.0 ± 12b	886.4 ± 213.9a	1391.7 ± 258.1b	17.4 ± 8.2a
PS2	3.9 ± 1.5b	22.9 ± 6.4c	431.1 ± 112.7b	569.7 ± 57.0c	14.5 ± 0.8a

*Note*:

*) Means followed by the same letter in the same column indicated not significantly different by DMRT at the level of 5%.

**Table 6:** The adlay growth and production on combination of fertiliser dosages and teak planting space treatments at 23 weeks after planting.

Interaction of fertiliser × planting space	Number of tiller	Number of sub-tiller	Number of grain/plant	Weight of 100 grain (g)	Shoot fresh weight (g)
F0PS4	6.7 ± 0.6 ab*)	46.7 ± 3.1 abcd	1559.0 ± 23.9 b	11.9 ± 0.8 a	762.2 ± 128.5 bc
F0PS3	4.3 ±1.5 bc	33.3 3± 7.1 cde	990.7 ± 101.0 c	12.4 ± 1.3 a	596.9 ± 81.0 cd
F0PS2	4.3 ± 1.2 bc	20.00 ± 2.6 e	384.7 ± 83.3 d	15.7 ± 1.0 a	373.7 ± 94.9 d
F2PS4	8.0 ± 1.7 a	63.67 ± 13.4 a	1917.3 ± 23.6 a	12.2 ± 1.9 a	1107.2 ± 121.0 a
F2PS3	5.3 ± 1.2 abc	43.33 ± 10.0 bcd	1569.3 ± 28.3 b	14.4 ± 0.7 a	1025.7 ± 274.5 ab
F2PS2	3.0 ± 0.0 c	19.67 ± 2.3 e	528.3 ± 25.2 d	14.2 ± 1.0 a	364.6 ± 57.7 d
F4PS4	8.0 ± 1.0 a	60.33 ± 8.5 ab	1650.0 ± 47.5 ab	13.5 ± 0.8 a	1154.7 ± 190.6 a
F4PS3	6.7 ± 2.9 ab	52.33 ± 18.8 abc	1615.0 ± 396.9 ab	25.5 ± 22.5 a	1036.8 ± 286.1 ab
F4PS2	4.3 ± 3.2 bc	29.00 ± 14.2 de	496.0 ± 62.6 d	15.7 ± 0.6 a	555.1 ± 185.6 cd

*Note*:

*) means followed by the same letter in the same column indicated not significantly different by DMRT at the level of 5%.
